# Transforming Medicine with Nanobiotechnology: Nanocarriers and Their Biomedical Applications

**DOI:** 10.3390/pharmaceutics16091114

**Published:** 2024-08-23

**Authors:** Arun Karnwal, Vikas Sharma, Gaurav Kumar, Amar Yasser Jassim, Aradhana Dohroo, Iyyakkannu Sivanesan

**Affiliations:** 1 Department of Microbiology, School of Bioengineering and Biosciences, Lovely Professional University, Phagwara 144411, India; arunkarnwal@gmail.com (A.K.); gauravkr01@gmail.com (G.K.); 2 Department of Molecular Biology and Genetic Engineering, School of Bioengineering and Biosciences, Lovely Professional University, Phagwara 144411, India; biotech_vikas@rediffmail.com; 3 Department of Marine Vertebrate, Marine Science Center, University of Basrah, Basrah 61004, Iraq; aalrshim@email.sc.edu; 4 School of Agricultural Sciences, Baddi University of Emerging Sciences and Technologies, Baddi 173405, India; dohrooaradhana@gmail.com; 5 Department of Environmental Health Science, Institute of Natural Science and Agriculture, Konkuk University, Seoul 05029, Republic of Korea

**Keywords:** targeted drug delivery, nanocarriers, novel nanostructures, precise therapeutics, nanobiotechnology, therapeutic approaches

## Abstract

Nanobiotechnology, at the intersection of nanotechnology and biology, represents a burgeoning field poised to revolutionize medicine through the use of advanced nanocarriers. These nanocarriers, endowed with distinctive physiobiological attributes, are instrumental in diverse therapeutic domains including drug delivery for microbial infections, cancer treatment, tissue engineering, immunotherapy, and gene therapy. Despite the transformative potential, several challenges hinder their efficacy, such as limited drug capacity, suboptimal targeting, and poor solubility. This review delves into the latest advancements in nanocarrier technologies, examining their properties, associated limitations, and the innovative solutions developed to address these issues. It highlights promising nanocarrier systems like nanocomposites, micelles, hydrogels, microneedles, and artificial cells that employ advanced conjugation techniques, sustained and stimulus-responsive release mechanisms, and enhanced solubility. By exploring these novel structures and their contributions to overcoming existing barriers, the article emphasizes the vital role of interdisciplinary research in advancing nanobiotechnology. This field offers unparalleled opportunities for precise and effective therapeutic delivery, underscoring its potential to reshape healthcare through personalized, targeted treatments and improved drug performance.

## 1. Introduction

The roots of nanotechnology and its biological counterpart, nanobiotechnology, trace back to pivotal milestones. Richard Feynman’s visionary 1959 lecture at Caltech laid the conceptual groundwork by proposing the manipulation of matter at the atomic and molecular scale [1]. However, it was Eric Drexler’s 1986 book, *Engines of Creation*, that catalyzed significant momentum in the field. Drexler provided a blueprint for precise molecular manipulation, propelling nanotechnology into the spotlight [2]. Essentially, nanotechnology involves engineering materials at the atomic, molecular, or macromolecular level to create structures with novel properties [3,4]. The goal is to enhance, modify, or impart new functionalities to these materials [5,6]. Progress in this domain hinges on understanding and harnessing the intricacies of cellular processes at the nanoscale [7]. Nanobiology emerged as a specialized branch of nanotechnology focused on biological systems. By integrating principles from nanotechnology, biology, and biochemistry, researchers developed innovative tools and materials [8]. Nanobiotechnology, a synergistic field, leverages the combined strengths of nanotechnology and biotechnology to optimize the properties and functions of nanomaterials or nanoparticles (NPs) [9]. A prime example is the use of functionalized nanoparticles (FNPs) for targeted drug or biomolecule delivery to specific tissues or organs, highlighting the transformative potential of this interdisciplinary approach [10]. The objective of the present review is to explore and evaluate the latest advancements in nanocarrier technologies, focusing on their unique properties, inherent limitations, and innovative solutions developed to address these challenges. This includes a thorough examination of their diverse biomedical applications, such as drug delivery, cancer treatment, tissue engineering, immunotherapy, and gene therapy, highlighting their transformative potential in these fields. Additionally, the objective is to identify the key challenges faced by nanocarriers—such as limited drug capacity, suboptimal targeting, and poor solubility—and discuss cutting-edge strategies employed to enhance their efficacy. Finally, it aims to emphasize the importance of interdisciplinary research in advancing nanobiotechnology, fostering collaboration across various fields to accelerate the development of more effective and personalized therapeutic treatments.

## 2. Rationale of Nanobiotechnology

### 2.1. The Remarkable Potential of Nanoparticles and Nanostructures in Biomedicine

Due to their extremely small size (1–100 nm) [11], nanoparticles (NPs) and other nanostructures have the unique capacity to pass through cell membranes, interact with organelles inside cells, and induce specific physiological reactions [12]. Nanostructures possess an innate capability that renders them crucial components in various biomedical domains, such as drug-delivery systems [13,14], imaging contrast agents [15], photothermal therapy, and advanced imaging technologies [16,17]. Table 1 displays a range of nanostructures and their utilization in the field of biomedicine.

### 2.2. Precision Targeting for Cancer Theranostics

Nanoparticles offer distinct benefits compared to conventional methods in the field of cancer theranostics (diagnosis and treatment). This benefit arises from the exceptional accuracy with which NPs selectively target particular sick cells or tissues, hence reducing the spread to adjacent healthy areas. The targeting process includes a combination of passive and active targeting mechanisms. Passive targeting exploits the enhanced permeability and retention (EPR) effect, which is characteristic of tumor tissues. Tumors have leaky vasculature and poor lymphatic drainage, allowing nanoparticles to accumulate preferentially in these areas [19,20]. On the other hand, active targeting involves the functionalization of nanoparticles with specific ligands or antibodies that recognize and bind to receptors overexpressed on the surface of diseased cells. These ligands can be proteins, peptides, aptamers, or small molecules that have a high affinity for the target receptors [21]. Upon binding, the nanoparticles are either taken up by the cells via receptor-mediated endocytosis or remain attached to the cell surface to deliver therapeutic agents or imaging probes. This dual targeting strategy enhances the precision of drug delivery, ensuring that the nanoparticles concentrate their therapeutic or diagnostic payloads specifically in diseased cells, thereby minimizing damage to healthy tissues and improving overall treatment efficacy.

Engineered targeted NPs are specifically designed to exclusively reach their intended destination, unlike conventional diffusing therapeutic agents or contrast agents. This targeted approach minimizes the risk of unwanted side effects and cytotoxicity in healthy cells that can occur with non-specific targeting [19,20]. By employing targeted administration, the necessary dosage for achieving therapeutic effectiveness is reduced, hence minimizing the likelihood of adverse reactions and enhancing patient outcomes [22].

### 2.3. Unveiling Cellular Mechanisms and Biomarker Discovery

Nanobiotechnology provides a robust framework for understanding complex biological pathways, signaling cascades, and the development of diseases. Nanobiotechnology significantly contributes to our comprehension of different diseases by improving the efficiency of identifying new biomarkers and revealing the mechanisms of therapeutic action [23,24]. Scientists can enhance their control over nanomaterials by manipulating and functionalizing them. This allows for the combination of nanomaterials with various bioactive molecules, such as nucleic acids, therapeutic drugs, photosensitizers, and enzymes. These combinations can be applied to specific biomaterials customized to meet specific needs [21]. Nanotechnology now offers exceptional opportunities in the fields of cancer diagnosis and prevention [25], antimicrobial medicines [26], and the reduction of various diseases [27].

## 3. Nanobiotechnology: A Cutting-Edge Intersection of Science and Medicine

**Figure 1:**
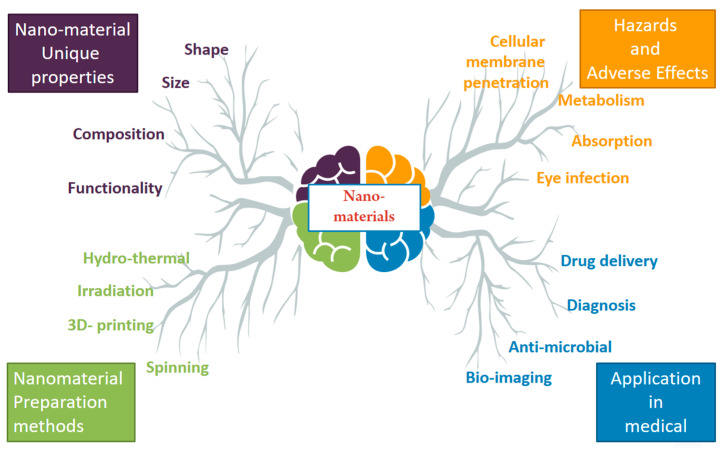
Nanomaterials properties, fabrication methods, and their application in bio-medicine.

Nanobiotechnology is a transformative change in the medical sector that involves the creation, advancement, control, and application of nanomaterials, particularly nanoparticles that are smaller than 100 nm [28]. This emerging discipline provides numerous possible remedies for diverse medical obstacles, principally by creating innovative nanocarriers and alternative drug delivery systems (DDS) [29]. Nanobiotechnology has had a notable impact by repurposing current medicinal molecules, improving their effectiveness, and perhaps addressing constraints linked to conventional delivery systems. Nanoparticles play a vital role in this process by shielding therapeutic molecules from enzymatic degradation and the reticuloendothelial system (RES) [30]. This protective mechanism enables prolonged circulation durations, resulting in enhanced bioavailability and heightened delivery efficacy to specific target locations [31]. The advent of nanobiotechnology signifies the merging of several fields, such as nanotechnology, biology, pharmacology, and physics. This interdisciplinary endeavor aims to foster the creation of innovative pharmaceutical nanomaterials and products that demonstrate enhanced effectiveness and greater flexibility. These applications cover a wide range, as seen in Figure 1:

- Drug delivery systems: Targeted delivery of therapeutic agents to specific cells or tissues, minimizing systemic side effects and maximizing therapeutic efficacy [31].
- Imaging: Development of contrast agents for improved diagnosis and monitoring of various diseases [32].
- Antimicrobial therapies: Combating antibiotic resistance through the development of novel antimicrobial nanomaterials [17].
- Anticancer therapies: Targeted delivery of chemotherapeutic agents to cancer cells, leading to increased efficacy and reduced off-target effects [33].
- In vitro diagnostics: Development of biosensors for rapid and accurate detection of diseases and biomarkers.

The field of nanobiotechnology is continually evolving, with researchers exploring and developing novel nanomaterials with diverse functionalities. These include nanoparticles, nanotubes, nanofibers, and other innovative structures, each offering unique advantages for specific applications. As research progresses, we can expect even more transformative advancements in nanomedicine, leading to personalized and effective treatments for various diseases [34].

### 3.1. Synthesis

Nanomaterials or nanostructures can be synthesized using both inorganic sources, such as silica, quantum dots, and metal nanoparticles, and organic materials like liposomes, micelles, dendrimers, and polymeric nanoparticles through various physical, chemical, or biological synthesis methods [35,36]. A wide range of nanostructures can be created with specific goals in mind, such as linking with specific drugs, achieving precise dispersion control, facilitating targeted transportation, and modifying for therapeutic purposes [37]. When nanoparticles (NPs) are functionalized with biomolecules or drugs, they can effectively evade immune cells, remain in the body for extended periods, disperse more efficiently, accumulate in target tissues at higher levels, prevent unintended spreading to nearby tissues, and release therapeutic agents or drugs in response to specific stimuli in a controlled manner over time. These properties make NPs ideal candidates for use as imaging or contrast agents, offering significant potential for therapeutic applications [18,38]. 

The fabrication of nanomaterials involves a complex interplay of scientific principles and technological advancements, with various methods offering distinct advantages and limitations. For example:

1. Physical methods: These techniques involve the application of external stimuli, such as laser ablation [39] or thermal decomposition [39], to induce nanoparticle formation. While these methods provide precise control over nanoparticle size and morphology, they may be limited in scalability and often require specialized equipment.
2. Chemical methods: Chemical reactions and self-assembly processes are employed to generate complex and intricate nanostructures [39]. These methods offer greater flexibility in tailoring the properties of nanoparticles but demand careful optimization and control of reaction parameters.
3. Biological methods: By leveraging biological systems, such as bacteria and plants, these methods present a sustainable and environmentally friendly approach to nanoparticle synthesis [39]. However, biological methods typically require longer processing times and may encounter challenges in achieving high yields and consistent quality.

**Figure 2:**
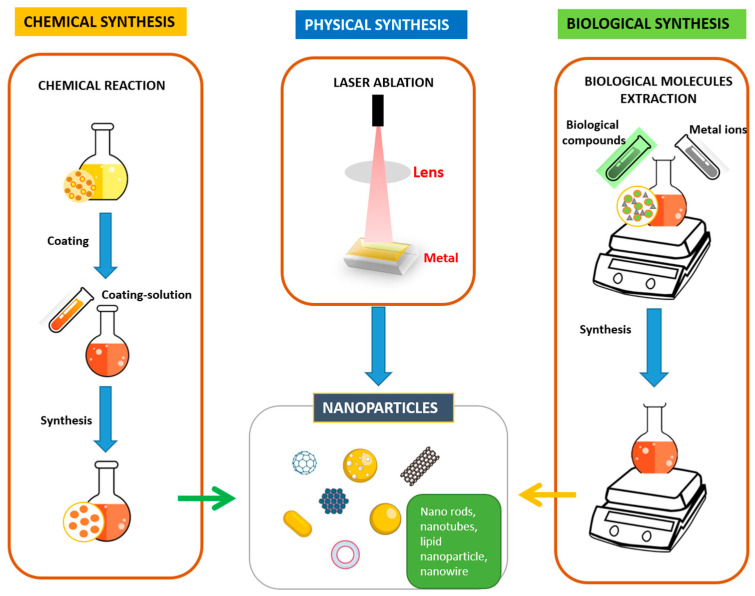
Nanomaterial synthesis: physical, chemical, and biological methods.

These methods, depicted in Figure 2, highlight the diverse approaches available for nanoparticle synthesis, each suited to specific applications and objectives.

### 3.2. Inorganic Nanoparticles: Paving the Way for Innovative Drug Design and Development

In drug design and development, inorganic nanoparticles (NPs), particularly magnetic nanoparticles (MNPs), have gained significant attention due to their unique physicochemical properties, including surface plasmon resonance (SPR), which is crucial for targeted drug delivery and improved therapeutic efficacy [40]. SPR arises from the collective oscillation of free electrons at the surface of metal nanoparticles, influencing their zeta potential, biological activity, and interactions with charged cellular surfaces. This phenomenon has been exploited for precise drug delivery, as evidenced by the incorporation of paclitaxel into modified iron oxide nanoparticles, which enables both superparamagnetic behavior and receptor-mediated targeting [41,42]. 

Beyond MNPs, other inorganic nanoparticles, such as nano-ceramide-graphene oxide (GO) hybrids and polymer-modified black phosphorus nanosheets, have demonstrated enhanced circulation, biodistribution, drug delivery, and anti-cancer effects [42,43]. These findings underscore the expanding field of nanobiotechnology, where inorganic nanoparticles are engineered to address the specific challenges of unconventional therapeutic candidates due to their nanoscale properties [44]. Among these nanoparticles, silver (Ag) has emerged as particularly noteworthy due to its distinctive biological properties and diverse potential applications [45]. The nanoscale size of silver enhances its ability to penetrate tissues, especially through compromised skin, and biomolecule-capped silver nanoparticles (AgNPs) display strong antibacterial activity and affinity for microbial and mammalian cells. AgNPs are highly effective in modulating inflammatory responses and preventing microbial growth, which has led to their extensive use in medical devices such as catheters, wound dressings, and implants to prevent infections [46]. Overall, inorganic nanoparticles present promising opportunities for the development of innovative therapeutics. Their unique properties and versatility offer significant potential for precise drug delivery, enhanced treatment efficacy, and improved patient outcomes. As research advances, further breakthroughs in the application of these nanoscale tools for disease treatment are anticipated [47].

### 3.3. Biocompatible and Biodegradable Nanoparticles for Drug Delivery: Exploring Beyond Polymers

Biocompatible and biodegradable nanoparticles (NPs) have emerged as a potent means of drug delivery, showcasing notable effectiveness through the utilization of materials such as chitosan (ChNPs), polyethylene glycol (PEG), polylactic acid (PLA), polycaprolactone (PCL), and polyacrylic acid (PAA) [48]. Nevertheless, the production of these nanoparticles frequently necessitates the use of several chemicals or solvents, which adds to the intricacy and may perhaps give rise to environmental apprehensions. In addition, certain polymeric nanoparticles (NPs) require the application of polymers or peptides, such as PEGylation [49], to enhance their biocompatibility and therapeutic benefits. However, these techniques are generally complex and time-consuming. In addition to polymers, lipid-based nanoparticles (NPs), namely, liposomes and nanoliposomes, have attracted considerable attention due to their simple manufacturing process and natural compatibility with living organisms [50]. Researchers have effectively created lipid-based nanoparticles (NPs) to transport different medications using techniques such as the thin-film dispersion approach. The utilization of this method has resulted in the enhancement of drug delivery by increasing the ability of the medication to be absorbed by the body and its ability to dissolve in water. This has been demonstrated through the encapsulation of brinzolamide using a stabilizer called hydroxypropyl-beta-cyclodextrin, as observed in a study conducted by Wang et al. [51].

Recent progress has also created opportunities for the development of precise delivery systems utilizing nanoliposomes. Researchers have improved the targeting specificity of nanoliposomes by combining mannose–cholesterol with PEG of different molecular weights [52]. Moreover, the distinctive composition of peptide–lipid nanoparticles, which integrates the beneficial characteristics of polymers and liposomes, has led to higher water solubility, drug dispersion, extended-release, and improved pharmacokinetics [53].

**Figure 3:**
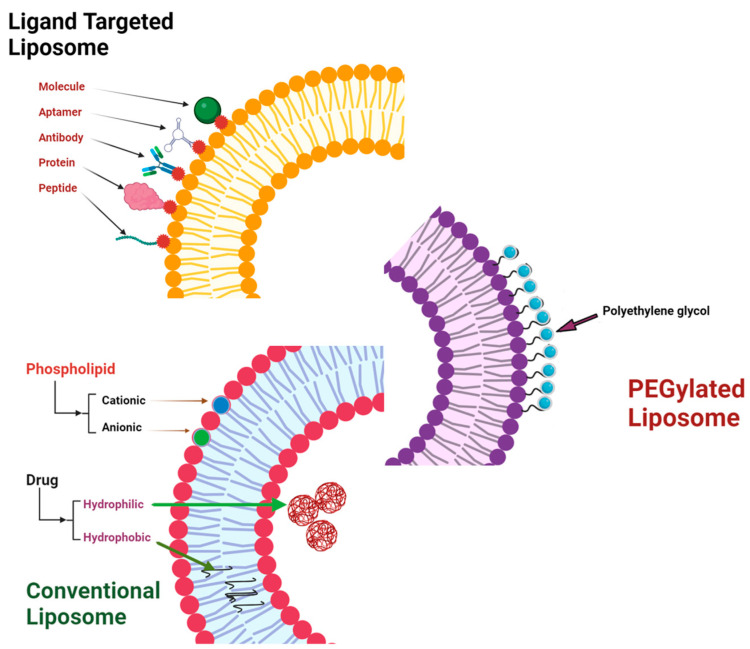
Liposomal structures and their modification through the incorporation of biomolecules, medications, or antibodies.

Liposomes are particularly notable among organic nanoparticles because of their remarkable flexibility. These formulations can be created with different sizes, compositions, and lipid molecules, allowing them to effectively transport a wide range of therapeutic agents, such as medicines (both water-insoluble and water-soluble), bioactive molecules, imaging agents, and photosensitizers (Figure 3). Due to their distinctive characteristic, they are well suited for transporting both hydrophobic and hydrophilic medicines [54]. In addition, liposomes can be customized to meet specific requirements by including surface changes such as PEG, target ligands, or leaving uncoated (Table 2). Liposomes have a notable characteristic of encapsulating several medications, contrast agents, and photosensitizers within a unified framework, enabling controlled release and precise delivery [55]. Liposomes have the potential to be a powerful tool for developing new and effective drug delivery systems. They are biocompatible and biodegradable, making them a promising alternative to polymeric nanoparticles. It is important to investigate these environmentally friendly options in drug delivery. Lipid-based nanoparticles (NPs), particularly liposomes, possess exceptional adaptability and the potential to deliver drugs to specific targets, making them a highly attractive area of research in drug delivery. By prioritizing these groundbreaking methodologies, we can establish a path for enhanced and accurate therapeutic interventions.

## 4. Emerging Frontiers in Nanomedicine: Nanocarriers for Enhanced Drug Delivery

**Figure 4:**
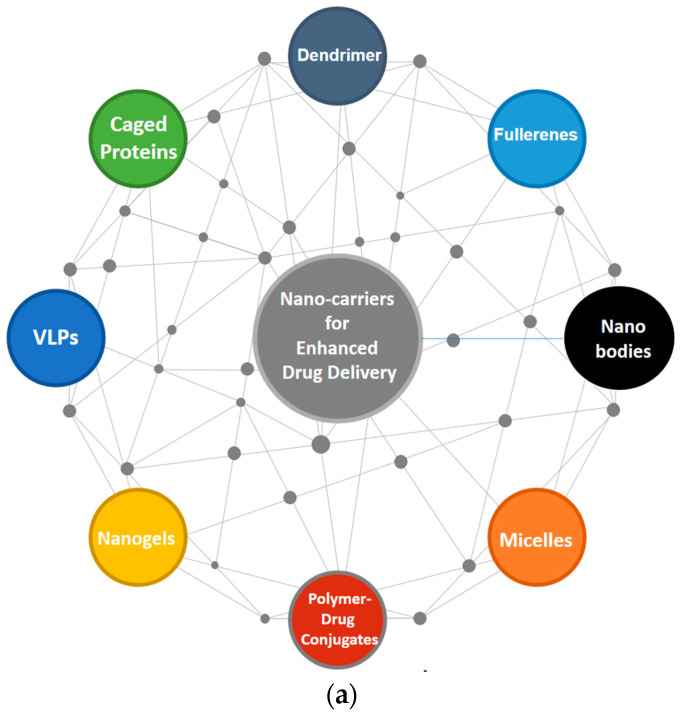

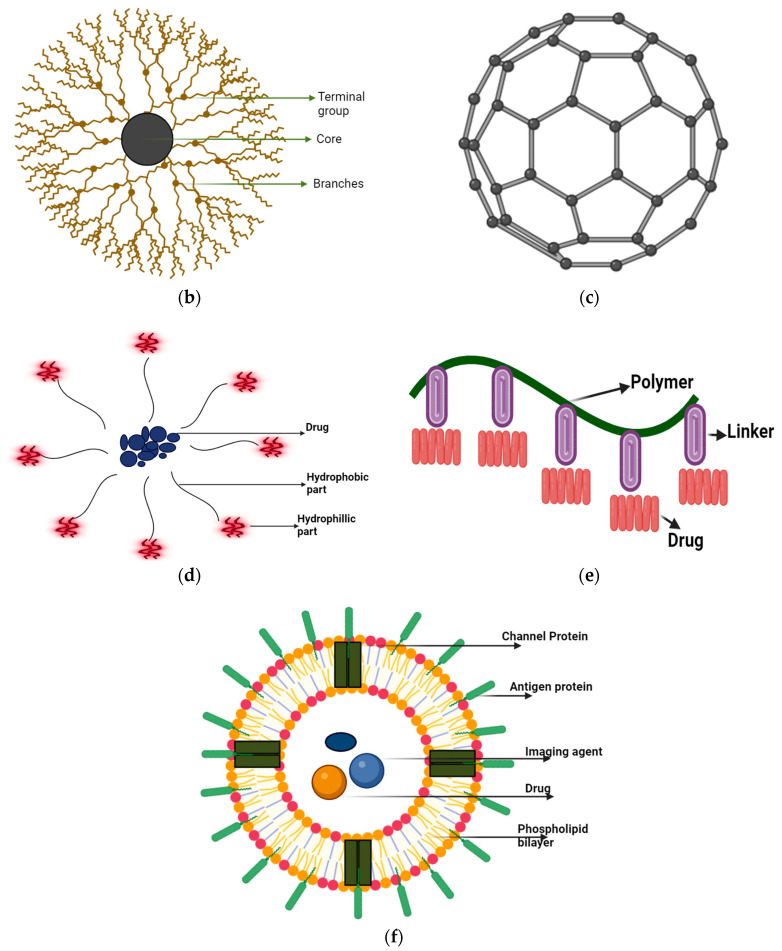
(**a**) Overview of varied nanocarriers used for improved drug delivery; (**b**) Dendrimers; (**c**) Fullerenes; (**d**) Micelles; (**e**) Polymer–drug conjugates; (**f**) Virus-like particles (VLPs).

The field of drug delivery has undergone a remarkable transformation with the advent of nanomedicine. Diverse nanocarriers (Figure 4), each with unique attributes and capabilities, offer unprecedented opportunities for the targeted and controlled release of therapeutic agents. This opens doors for the development of novel therapeutic strategies with improved efficacy and reduced side effects. Table 3 presents the diverse nanocarriers used for enhanced drug delivery and their advantages and disadvantages.

### 4.1. Dendrimers

These meticulously designed monodisperse macromolecules possess a compact core adorned with numerous functional groups [58]. This architecture facilitates high drug loading and controlled release, rendering them ideal for delivering anticancer drugs, gene therapy vectors, and imaging probes. Their nanoscale size, homogeneity, and enhanced cellular uptake further contribute to their effectiveness in targeted drug delivery [59].

### 4.2. Fullerenes

Caged carbon allotropes with hollow interior fullerenes offer a unique platform for drug encapsulation and controlled release [60]. Their functionalized surfaces allow for targeted delivery, while their responsiveness to specific stimuli, such as pH changes or light exposure, enables the triggered release of drugs. This tunability makes them promising candidates for delivering anticancer drugs, antibiotics, and imaging agents.

### 4.3. Nanobodies

Single-domain fragments of antibodies and nanobodies offer superior solubility, stability, and tissue penetration compared to their conventional counterparts. This translates to enhanced tumor targeting and deeper tissue penetration, making them valuable tools for targeted drug delivery [61]. Additionally, their ability to be readily conjugated with various drugs and biomolecules opens doors for developing multifunctional nanocarriers for theranostics, high-quality imaging, and anticancer therapy [62].

### 4.4. Micelles

Dynamic self-assembled structures formed by amphiphilic block copolymers and micelles possess hydrophilic heads and hydrophobic cores. This configuration allows for efficient encapsulation of both hydrophobic and hydrophilic drugs, making them versatile drug delivery systems. Furthermore, their prolonged circulatory retention and resistance to phagocytosis contribute to their therapeutic efficacy [63].

### 4.5. Polymer–Drug Conjugates

These conjugates are formed by covalently linking drugs to polymer chains, resulting in a single entity with improved properties. This approach enhances drug solubility, stability, and retention within the body, enabling controlled release at specific disease sites [64]. Moreover, the ability to design polymer–drug conjugates for triggered release further optimizes therapeutic efficacy and minimizes systemic side effects.

### 4.6. Virus-like Particles (VLPs) and Caged Proteins (CPs)

Biomimetic nanocarriers mimicking viral structures without viral genomes, VLPs, and CPs offer inherent biocompatibility and reduced risk of immune responses. This makes them attractive candidates for drug delivery, particularly in the context of cancer treatment. Additionally, their ability to elicit immune responses holds promise for vaccine development and cancer immunotherapy [65].

### 4.7. Self-Assembled Protein Nanoparticles

Biocompatible and versatile, these nanoparticles are derived from various natural proteins like collagen, soy, and albumin [66]. Their inherent biodegradability and ease of functionalization allow for diverse applications, including tissue regeneration, wound healing, and drug delivery [67]. Notably, albumin-based nanoparticles like Abraxane demonstrate their potential in delivering paclitaxel for cancer treatment, while virus-like protein-based vaccines highlight their application in combating diseases like HIV.

### 4.8. Nanogels

These gel-like polymeric nanocarriers offer several advantages for drug delivery, including ease of preparation, diverse carrying capacity, minimal cargo efflux, and applicability in various fields [68]. Their potential extends beyond their established applications in tissue regeneration, wound healing biomaterials, bioelectronics, and biochemistry, holding promise for vaccines, nucleic acid delivery, and immunotherapy.

**Table 3:** Exploring Diverse Nanocarriers for Enhanced Drug Delivery [64,66,69].

Nanocarrier	Properties	Drug Conjugation	Advantages	Disadvantages	Potential Applications
Dendrimers	- Branched, monodisperse polymers - High drug loading capacity - Tailorable surface functionalization	Covalent or non-covalent binding	- Targeted delivery - Controlled release - Enhanced bioavailability	- Complex synthesis - Potential immunogenicity	- Cancer therapy - Gene delivery - Antiviral therapy
Fullerenes	- Carbon cages with unique chemical properties - Large internal cavity - Biocompatibility challenges	Non-covalent interactions (supramolecular assembly)	- High drug loading capacity - Protection from degradation - Multifunctional capabilities	- Limited solubility - Difficult functionalization - Potential toxicity	- Cancer therapy - Photodynamic therapy - Antioxidant therapy
Nanobodies	- Single-domain antibodies - Small size (~12 kDa) - High affinity and specificity	Genetic fusion or chemical conjugation	- Targeted delivery to specific receptors - Reduced off-target effects - Deep tissue penetration	- Expensive production - Limited drug loading capacity - Potential immunogenicity	- Cancer therapy - Immunosuppression - Targeted diagnostics
Micelles	- Self-assembled amphiphilic molecules - Core–shell structure for hydrophobic/hydrophilic drugs - Dynamic and versatile	Encapsulation within the core or conjugation to shell	- Enhanced bioavailability - Controlled release - Targeted delivery through surface modification	- Potential leakage of drugs - Limited drug loading capacity for some molecules	- Drug delivery for cancer, antibiotics, genes, etc. - Imaging agents - Cosmetics
Polymer-Drug Conjugates	- Covalent attachment of drug to polymer carrier - Tailorable release profiles - Improved solubility and targeting	Direct conjugation or linker molecules	- Controlled release - Prolonged circulation - Targeted delivery through polymer characteristics	- Complex synthesis and characterization - Potential toxicity of carrier system	- Cancer therapy - Antiviral therapy - Gene delivery
Virus-Like Particles (VLPs)	- Non-replicating viral mimics - Highly organized and stable structures - Efficient cellular uptake	Genetic engineering or encapsulation	- Targeted delivery to specific cell types - Multivalent presentation of drugs - Immunostimulatory properties	- Potential safety concerns (pre-existing immunity) - Complex production processes	- Vaccines - Gene therapy - Cancer therapy
Caged Proteins (CPs)	- Engineered proteins that release drugs upon external stimuli (light, pH, etc.) - High spatial and temporal control - Reduced systemic exposure	Non-covalent encapsulation or protein fusion	- Targeted delivery - Controlled release - Reduced side effects	- Complex protein engineering - Potential immunogenicity - Limited payload capacity	- Cancer therapy - Pain management - Neurodegenerative diseases
Self-Assembled Protein Nanoparticles	- Protein-based nanoparticles formed through physical interactions - Biocompatible and biodegradable - Multifunctional capabilities	Protein engineering and self-assembly	- Targeted delivery - Controlled release - Combination therapy potential	- Limited drug loading capacity - Potential heterogeneity in size and structure	- Drug delivery for various diseases - Imaging agents - Tissue engineering
Nanogels	- Cross-linked polymer networks with tunable properties - High drug loading capacity - Responsive release mechanisms	Encapsulation, conjugation, or intra-gel crosslinking	- Controlled release - Targeted delivery - Multifunctional capabilities	- Potential leakage of drugs - Complex synthesis and characterization	- Drug delivery for various diseases - Cell therapy - Imaging agents

## 5. Multifaceted Bio-Impact of Nanoparticles: From Cellular Disruption to Therapeutic Potential

Nanoparticles (NPs) demonstrate a complex and multifaceted array of effects within biological systems, showcasing their significant influence on various cellular processes [52]. One of their primary actions involves the disruption of cellular homeostasis by inducing nucleic acid denaturation, leading to altered mitochondrial membrane potential and impaired function of lipids, proteins, and mitochondria, as mentioned in Table 4 and Table 5 [70]. This detrimental effect is further amplified by the generation of reactive oxygen species (ROS) and subsequent oxidative stress [71], culminating in the initiation of apoptosis via cytochrome-c expression and intracellular cation deposition [72]. Additionally, NP-induced inflammation underscores their diverse impact on various biological pathways [73].

Beyond intracellular disruption, NPs exhibit a proclivity for interacting with sulfhydryl groups and inactivating crucial metabolic enzymes within the mitochondrial transport chain, further compromising cellular integrity [74]. Their affinity for plasma membrane proteins and the phosphorus moieties of DNA also plays a detrimental role, hindering DNA replication processes and potentially contributing to genotoxicity [75]. Notably, silver nanoparticles (AgNPs) have been shown to specifically displace Zn^2+^ and Ca^2+^ ions, adding another dimension to their bio-interaction portfolio [76].

However, the impact of NPs extends beyond these intracellular effects. Their ability to disrupt biofilm frameworks and microbial structures offers promising applications in antimicrobial therapy [77]. Additionally, their versatility as drug carriers allows for targeted delivery of therapeutic agents to specific sites or tissues, leveraging the enhanced permeability and retention (EPR) effect to ensure prolonged drug availability at the desired location [78]. Furthermore, their potential to induce endocytosis facilitates the development of effective and targeted therapy strategies [79].

For a more detailed understanding of the intricate mechanisms underlying the bio-interactions of magnetic nanoparticles (MNPs), we recommend referring to our previous publication, which provides a comprehensive elucidation of their specific actions.

**Table 4:** Nanoparticle Design for Therapeutic Applications: Exploring the Intersection of Size, Shape, and Cellular Disruption [52,71,72,73].

Aspect	Description	Implications for Therapeutics
Size	- Typically ranges from 1 to 1000 nm. - Influences biodistribution, cellular uptake, and clearance.	- Smaller particles (<100 nm) generally penetrate tissues more effectively. - Size affects renal clearance and accumulation in specific organs.
Shape	- Common shapes include spherical, rod-like, and disk-shaped. - Shape affects cellular uptake and tissue distribution.	- Spherical nanoparticles often have better uniformity and stability. - Rod-like and disk-shaped particles can enhance cellular internalization and target specific tissues.
Surface Chemistry	- Modifications include coating with PEG, targeting ligands, or charge adjustments. - Impacts stability, circulation time, and targeting.	- PEGylation can increase circulation time and reduce immunogenicity. - Targeting ligands improve specificity to target cells or tissues.
Cellular Disruption	- Refers to the ability of nanoparticles to interact with or disrupt cellular processes. - Can include endocytosis, apoptosis induction, or cell membrane disruption.	- Enhances drug delivery efficiency by facilitating cellular uptake. - Can be used to trigger controlled cell death in cancer therapy.
Biocompatibility	- Compatibility with biological systems, including low toxicity and minimal immune response.	- Essential for safe and effective therapeutic applications. - Ensures minimal adverse effects and better patient outcomes.

**Table 5:** Effect of Nanoparticles on Cellular Disruption and Therapeutic Potential [78].

Cellular Disruption Mechanism	Nanoparticle Characteristics	Therapeutic Potential	Challenges and Considerations
Membrane Disruption	Sharp edges or high surface area, Functionalized with hydrophobic groups, High surface charge	Targeted delivery of drugs directly to diseased cells, Disruption of bacterial or viral membranes, Enhanced gene delivery	Off-target effects on healthy cells, Potential for inflammation and cytotoxicity, Difficulty in controlling targeting specificity
Oxidative Stress	Metal oxide NPs, Carbon nanotubes, Some polymer NPs	Induction of cell death in tumors, Modulation of immune response, Antibacterial activity	Damage to healthy tissues due to reactive oxygen species (ROS) Potential for genotoxicity and carcinogenicity, Need for antioxidant co-administration
Protein Corona Formation	Protein adsorption on nanoparticle surface, Changes in nanoparticle size and surface charge	Modulation of cellular signaling pathways, Enhanced drug delivery through protein-mediated recognition, Potential for unintended interactions with biological systems	Difficulty in predicting and controlling protein composition, Can affect targeting specificity and cellular uptake
Mitochondrial Dysfunction	Accumulation of NPs in mitochondria, Interference with electron transport chain, Release of pro-apoptotic factors	Selective cytotoxicity to cancer cells, Induction of programmed cell death, Need for precise targeting to avoid damage to healthy mitochondria	Potential for off-target effects and systemic toxicity, Difficulty in designing NPs with specific mitochondrial affinity
DNA Damage	Genotoxic NPs (e.g., some metal ions), Induction of reactive oxygen species, Disruption of DNA repair mechanisms	Killing of cancer cells by inducing mutations, Targeting specific genetic mutations for therapy	High risk of genotoxicity and potential for long-term health effects. Need for strict safety evaluation and controlled delivery
Endoplasmic Reticulum Stress	NPs cause protein misfolding and aggregation, disrupt calcium homeostasis, and activate unfolded protein responses	Induce apoptosis in cancer cells, Suppress tumor growth and angiogenesis	The potential for triggering unintended cell death pathways Can lead to inflammation and organ toxicity
Autophagy Dysregulation	NPs interfering with autophagy machinery, Accumulation of damaged proteins and organelles, Induction of cell death or survival	Selective killing of cancer cells with impaired autophagy, Potential for neurodegenerative diseases and other pathologies	Difficulty in controlling the extent and direction of autophagy modulation, Risk of triggering unintended cellular responses

## 6. Therapeutic Applications of Nanobiotechnology

### 6.1. Antimicrobial Therapy

**Figure 5:**
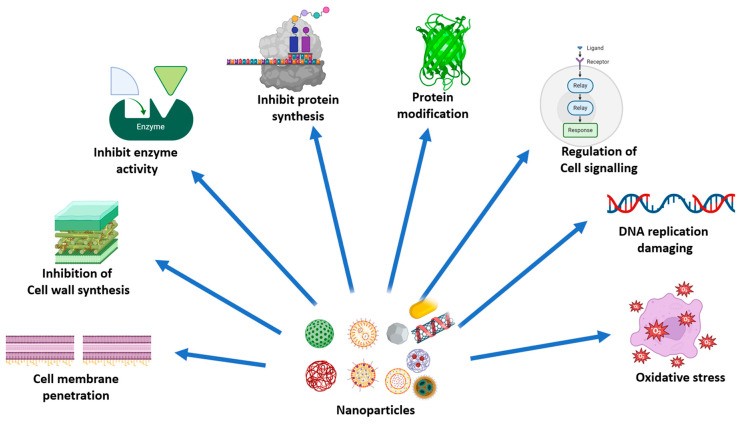
Various strategies employed for the antibacterial activity of nanoparticles [82].

The emergence of multidrug-resistant (MDR) microbial strains poses a significant threat to global health, necessitating the development of novel and effective therapeutic strategies. Nanoparticles (NPs) have emerged as promising candidates in this battle, offering unique properties and functionalities that enhance the efficacy and safety of conventional antimicrobial agents (Figure 5).

**Polymer-based NPs:** These versatile carriers provide sustained release of antibiotics, improving their therapeutic effectiveness while reducing the risk of side effects. A notable example is Lipoquin, a liposomal formulation of ciprofloxacin used for respiratory infections. Lipoquin effectively delivers the antibiotic over an extended period, eliminating the need for high-dose or combination therapies [80].

**Immune cell-based nanoformulations:** These innovative systems harness the inherent targeting capabilities of immune cells to deliver antimicrobial drugs directly to infected sites. This targeted approach minimizes systemic exposure to the drug, further reducing potential toxicity. An example is the use of macrophage-encapsulated vancomycin nanoparticles for treating severe MRSA infections [81].

**Liposomes:** These spherical vesicles composed of lipids offer a biocompatible platform for encapsulating various antifungal drugs, including amphotericin B. Liposomal formulations significantly reduce the cytotoxicity of amphotericin B, enabling its effective use against fungal infections like neutropenia, histoplasmosis, and even viral infections [83,84].

**Chitosan nanoparticles (ChNPs):** The effectiveness of these biocompatible and biodegradable NPs against multidrug-resistant infections, such as oral microorganisms and Neisseria gonorrhoeae, has been encouraging. It has been demonstrated that ChNPs can efficiently kill bacteria like *Staphylococcus* species, *Enterococcus* species, and *Candida* species that are both planktonic and biofilm-forming [85].

**Silver nanoparticles (AgNPs):** These potent antimicrobial agents have been incorporated into various materials, including implants and wound dressings, to combat infections. Silverline, Verigene, Acticoat, and Endorem are some of the commercially available products utilizing AgNPs for wound infection control [81]. Interestingly, nanocomposites combining silver, fluoride, and chitosan have exhibited remarkable antimicrobial properties against *Enterococcus* spp. and *Candida* spp., with their size playing a crucial role: smaller particles (<10 nm) displayed higher toxicity to macrophages [86].

**Polymeric NPs with microcin J25 AMPs:** It has been established that these new nanoparticles, which were produced by conjugating chitosan with microcin J25 antimicrobial peptides, have demonstrated substantial bactericidal effects against tetracycline-resistant *E. coli* K88 and MRSA [87]. These effects are dose-dependent. Furthermore, these nanoparticles had no harmful effects on Caenorhabditis worms, emphasizing their encouraging prospects for use in medical treatments [88].

### 6.2. Tissue Regeneration

Driven by the challenges of delayed wound healing, multidrug-resistant (MDR) infections, and compromised immune states, researchers are pursuing innovative strategies in the field of tissue engineering (TE) and regeneration (TR) [7]. These efforts extend beyond conventional bandages and drugs, embracing the potential of functionalized nanoparticles (NPs) to revolutionize therapeutic approaches. NPs offer a unique platform for tissue regeneration by interacting with cells, the environment, and microbes with enhanced efficiency. Their ability to create an oxygen-deprived microenvironment conducive to regeneration stimulates responses from stem cells and enzymes, accelerating the healing process [89]. Diverse fabrication methods, including electrospinning and coaxial techniques, are utilized to create nanofibrous scaffolds, nanogels, hydrogel scaffolds, thread-based patches, and sponge scaffolds [90].

**Figure 6:**
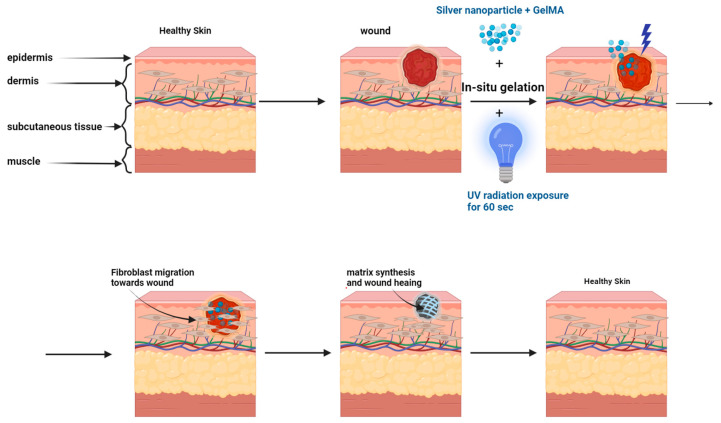
Potential scaffolds for wound healing: GelMA gels encapsulating silver nanoparticles.

The antimicrobial and wound-healing properties of AgNPs have led to their extensive application in various materials (Figure 6). Incorporating AgNPs into gels, hydrogels, fiber meshes, or polymeric membranes has yielded unique bandages with potent antibacterial activity [91]. Functionalized nanogels and nano-meshes containing AgNPs, growth hormones, antibiotics, and enzymes hold great promise as wound dressing systems, enhancing immune response, controlling MDR microbial growth, and promoting wound healing [92]. Concerns about cytotoxicity and the need for enhanced therapeutic efficacy continue to drive the development of innovative targeted drug delivery systems. Researchers are exploring a diverse range of materials and techniques beyond traditional polymers and liposomes to achieve more precise and effective therapies.

### 6.3. Nanofibers for Targeted Delivery

Electrospinning, a versatile technique for fabricating nanofibers from various polymers, holds immense promise for targeted drug delivery. These nanofibers can encapsulate and deliver enzymes, anticancer drugs, and antimicrobial drugs with high efficiency, as demonstrated by a group of researchers [93]. Coaxial electrospinning, explored by scholars [94], further expands this potential by enabling the fabrication of sophisticated nanostructures like nanocapsules, nanotubes, and nanochannels, allowing for precise and controlled release of therapeutic agents.

The field of targeted drug delivery is undergoing a revolution, with researchers exploring materials and techniques beyond traditional polymers and liposomes. These advancements offer promising avenues for developing more effective and precise therapies with reduced cytotoxicity and enhanced therapeutic efficacy. Future research will likely focus on further refining these novel approaches and exploring their applications in various therapeutic areas.

## 7. Organic Nanostructures and Diverse Applications

Organic nanostructures like PLGA, PVA, polyamide, PMMA, and PEVA are attracting significant interest due to their ability to entrap and co-deliver diverse therapeutic agents. This versatility provides a platform for developing novel treatment strategies for various diseases [95].

### 7.1. Advances in Organic Nanoparticles for Targeted Cancer Therapy

In recent years, organic nanoparticles (NPs) have witnessed a surge in research for their potential in targeted cancer therapy, revolutionizing drug delivery systems. Nanostructures composed of PEG-PLA and PEG-PLGA are particularly promising for delivering bioactive molecules and therapeutic drugs [96]. These materials offer exceptional stability and controlled release, contributing significantly to their effectiveness in cancer treatment. Additionally, carbon-based materials like graphene oxide (GO), quantum dots (QDs), and nanotubes have garnered attention due to their unique dimensional properties and potential anticancer effects [97]. The versatility of these nanomaterials opens new avenues for targeted drug delivery and innovative therapeutic approaches in cancer management.

Among PEG-based NPs, precisely designed formulations like PEG-platinum and PEG-Ag nanostructures incorporate both hydrophilic and hydrophobic components, enhancing their suitability for drug delivery [98]. Furthermore, PEG-PLA copolymer-based NPs have been engineered for targeted delivery of specific anticancer drugs like Capecitabine and hydrophobic platinum compounds.

Recent advancements in PEG-based NPs include the development of PEG conjugated with beta-Cyclodextrin, demonstrating enhanced capabilities for carrying and delivering anticancer drugs like Doxorubicin and Sorafenib [98,99]. Moreover, a versatile modified PEG–Cyclodextrin complex has been explored for applications in immunotherapy and diabetic therapy, showcasing the exceptional potential of these nanocarriers [100]. Additionally, PEG-PCL copolymer, formed by conjugating PEG with beta-Caprolactone, has been meticulously engineered for targeted delivery of hydrophobic drugs or biomolecules like cytokines against various cancer types [101]. This ongoing research highlights the continuous efforts to tailor nanoparticle formulations for specific therapeutic applications. While Polyethylene Glycol (PEG) and Polylactic Acid (PLA) remain dominant in the realm of nanoparticle-based drug delivery, researchers are venturing beyond these traditional polymers to explore a wider range of materials with diverse functionalities, particularly in the fight against cancer. This exploration holds immense potential for developing more effective and targeted therapies.

### 7.2. Polysaccharides for Dermatological and Photodynamic Therapy

Polysaccharides, such as polygalactose, hyaluronic acid, and chitosan, have emerged as promising alternatives due to their biocompatibility and unique properties. For example, Ref. [102] developed chitosan nanoparticles encapsulating the natural anticancer agent quercetin for dermatological applications, demonstrating effective protection against UV radiation. Similarly, Ref. [103] successfully utilized self-assembled hyaluronic acid micelles encapsulating hydrophobic photosensitizers for photodynamic therapy against cancer. This approach provides controlled delivery with enhanced efficacy through redox-responsive kinetics.

### 7.3. Glycopolymer-Functionalized Nanoparticles for Targeted Delivery

Functionalizing nanoparticles with glycopolymers presents another exciting avenue for targeted delivery. There are one hundred twelve designed glycopolymer-functionalized nanoparticles for the targeted release of biomolecules or drugs to cancer sites, particularly for the delivery of hydrophobic drugs. This strategy offers enhanced targeting and improved therapeutic outcomes.

### 7.4. Liposomes and Beyond: Vincristine-Sulfate and Lipoplatin

Liposomes, especially those smaller than 120 nm, offer significant advantages due to their prolonged circulation, reduced cytotoxicity, and accumulation at diseased target sites through the enhanced permeability and retention (EPR) effect [104]. This enhanced targeting capability opens doors for a wider range of applications.

Beyond liposomes, lipid-based and polymeric formulations like Vincristine-Sulfate have shown promising results against lymphocytic leukemia. Additionally, Lipoplatin, a functionalized derivative of Cisplatin, exhibits reduced cytotoxicity and has been approved for the treatment of rare cancers [105].

## 8. Expanding the Repertoire of Nano-Drugs

Beyond AgNPs, other nanoparticles like selenium nanoparticles (SeNPs) are also showing promise in cancer treatment [106,107]. Studies have demonstrated the in vitro cytotoxic effects of SeNPs against oral squamous cell carcinoma and colorectal adenocarcinoma cells [108,109]. These findings contribute to the growing repertoire of nano-drugs with potential for clinical application.

### 8.1. Clinical Advancements: From Genexol-PM^®^ to Myocet™

Recent advancements in nanomedicine have led to the development of several FDA-approved nano-drugs for cancer treatment. These include polymeric micelle formulations like Genexol-PM^®^, liposomal doxorubicin (Doxil^®^), and non-PEGylated liposomal doxorubicin Myocet™ [110]. These advancements demonstrate the continuous evolution and clinical translation of nanotechnology in cancer therapy (Table 6 and Table 7).

### 8.2. MNPs: Enhanced Delivery and Targeting

Magnetic nanoparticles (MNPs) offer another promising approach to targeted drug delivery, as mentioned in Table 7 [115,116]. AgNPs derived from *Setaria verticillata* extracts have been successfully used as carriers for anti-cancer drugs, demonstrating their potential in drug delivery systems (DDSs) [117]. Additionally, dual delivery of doxorubicin and alendronate using AgNPs has shown high efficacy against cancer cells, highlighting the potential of this approach for combination therapies [118].

### 8.3. Active Targeting and Vehicle Redesign

Researchers are actively exploring strategies for active targeting of cancer cells using AgNP-based DDSs. This involves modifying the nanoparticle surface with specific ligands that bind to cancer cell receptors, enabling targeted delivery and enhanced therapeutic efficacy [119]. Furthermore, vehicle redesign has been utilized to modulate drug cargo distribution within packets, achieving sustained and long-term delivery of anti-cancer drugs [120]. This approach offers a promising avenue for achieving sustained cytotoxic effects against cancer cells.

### 8.4. Apoptotic Pathways and Caspase-3 Quantification

The apoptotic pathway, a crucial mechanism of cell death, can be triggered by various stimuli, including MNPs, UV and gamma rays, or oxidative stress induced by reactive oxygen species (ROS). AgNPs, anti-cancer drugs, or radiation can initiate this pathway through a series of events, which can be monitored by quantifying caspase-3 activity [121,122]. This provides valuable insights into the mechanisms of action of these therapeutic interventions. Nanomedicine offers a revolutionary approach to cancer treatment, with AgNPs and other nanoparticles demonstrating significant potential for targeted drug delivery and enhanced therapeutic efficacy [31]. Ongoing research and development efforts are paving the way for the translation of these promising therapies into clinical practice.

## 9. Factors Affecting Properties and Applications

*Modulation of Surface Chemistry and Functionalization of Nanomaterials: A Comparative Analysis*

The diverse methods employed in the synthesis of nanomaterials significantly impact their surface chemistry, ultimately affecting their biocompatibility, functionality, and potential applications.

### 9.1. Biological Synthesis: Advantages and Limitations

Biological synthesis provides an uncomplicated and eco-friendly method for producing nanomaterials. By using plant extracts or microbial enzymes as reducing and capping agents, this approach is often a more cost-effective alternative compared to other techniques [36]. Additionally, biomolecules present in biological systems can stabilize the nascent nanoparticles, enhancing their stability and dispersibility [123].

However, the inherent lack of precise control in biological synthesis can result in nanoparticles with diverse sizes and surface chemistries, potentially compromising their efficacy for specific applications. Moreover, the presence of phytochemicals or microbial enzymes can degrade biomolecules like drugs or photosensitizers during bioreduction, necessitating additional chemical modification steps for functionalization [124,125].

### 9.2. Chemical Synthesis: Versatility and Control at a Cost

Chemical methods provide a robust and versatile platform for tailoring the surface chemistry and functionality of nanomaterials. Through careful selection of precursors and reaction parameters, researchers can synthesize nanoparticles with specific sizes, shapes, and compositions, facilitating their application in diverse fields [126].

Chemical synthesis also offers superior control over the loading of bioactive molecules onto the nanomaterials’ surface. By employing various chemical conjugation strategies, scientists can attach antibodies, targeted ligands, and photosensitizers with high accuracy, enabling tailored therapeutic and diagnostic applications [114].

Despite its advantages, chemical synthesis often requires specialized equipment, hazardous chemicals, and meticulous control over reaction parameters, significantly increasing both cost and complexity [127]. Additionally, concerns regarding the environmental impact of chemical reagents and the potential for residual toxicity in the final product necessitate careful consideration and robust environmental safety protocols.

### 9.3. Physical Synthesis: High Precision with High Cost and Complexity

Physical methods offer the highest degree of control over the size, shape, and surface morphology of nanoparticles. Techniques like laser ablation and pulsed laser deposition allow for precise tailoring of nanomaterial properties, making them suitable for specialized applications in electronics, optics, and energy storage [128].

However, physical methods typically require expensive and sophisticated equipment, limiting their accessibility and applicability [129]. Moreover, the functionalization of physically synthesized nanomaterials often necessitates additional steps, further increasing the complexity and cost of the overall process.

The ideal approach for nanoparticle synthesis depends on the desired properties, intended application, and available resources. Biological methods offer a simple and cost-effective option for achieving basic functionalities, while chemical synthesis provides greater control and versatility, albeit at a higher cost and environmental impact [130]. Physical methods, while offering the highest degree of precision, are often limited by their high cost and complexity.

Future research should focus on developing more sustainable and cost-effective synthesis methods that combine the advantages of different techniques while mitigating their limitations [131]. Additionally, further studies are required to fully understand the long-term biological and environmental implications of each method, ensuring responsible development and application of nanotechnology.

## 10. Deciphering the Intricacies of Nano–Bio Interactions: Unveiling the Role of Functionalization

The intricate interplay between nanoparticles (NPs) and biological systems is profoundly influenced by the parameters of functionalization, which involves modifying physiochemical properties through the addition or conjugation of biomolecules, reduction, or stabilization with various materials [113]. This meticulously crafted process significantly alters surface chemistry, chemical groups, and composition, ultimately impacting antimicrobial activities, biological uptake, and even cytotoxicity [132].

Surface functionalization can be readily achieved through diverse strategies, including PEGylation, coating with polymers like chitosan, antibodies, peptides, folic acid, biotin molecules, or biomolecules extracted from plants. Alternatively, NPs can be deposited onto such polymers to achieve desired modifications [133]. However, even minor variations in surface chemistry can significantly influence the biological and physiochemical properties of NPs.

Substantial research highlights the positive impact of functionalization on various biological actions. For instance, NPs modified with PEG, polysaccharides like dextran, or oligosaccharides like chitosan have demonstrated enhanced biological activity [134]. Similarly, mesoporous silica-modified NPs exhibit improved stability and disparity.

The significance of functionalization is further underscored by comparing the impacts of capped and uncapped nanoparticles (NPs). The study conducted by [98,135] found that Starch-capped copper nanoparticles (S-CuNPs) showed mild toxicity in mouse embryonic fibroblasts (3T3L1) cells without causing any alterations in cell morphology. In contrast, uncapped copper nanoparticles (CuNPs) exhibited much higher cytotoxicity. Similarly, the presence of chitosan-coated silver nanoparticles (Cs-AgNPs) at concentrations as low as 10 µg/mL [136] causes notable toxicity and changes in the cellular structure of RAW264.7 macrophages.

Moreover, the process of functionalization can impact the selectivity of nanoparticles. The cytotoxic and genotoxic effects of Selenium NPs (SeNPs) were significantly higher when they were functionalized with poly-l-lysine (PLL) compared to SeNPs coated with polyacrylic acid (PAA) and polyvinylpyrrolidone (PVP). This emphasizes the significance of appropriate functionalization for achieving targeted effects [137]. These findings illuminate the critical role of functionalization in dictating the biological fate and targeting capacity of NPs. By meticulously tailoring surface chemistry and composition, researchers can unlock the full potential of NPs while mitigating potential risks associated with their interactions with biological systems. As research in this field continues to flourish, we can anticipate even more precise and targeted NP-based solutions for diverse applications in medicine, environmental remediation, and beyond.

## 11. Factors Influencing Their Biological Fate and Impact

The biological journey of nanoparticles (NPs) is a complex dance influenced by a diverse array of environmental factors. Temperature, pH, oxygen levels, the intricate biochemical milieu, and the presence of pathogens or toxins all play intricate roles in shaping the biological properties and ultimate fate of these nanoscale entities [138,139].

Temperature: Minor fluctuations in temperature can significantly impact the stability, aggregation behavior, and biodistribution of NPs [140]. For instance, elevated temperatures can enhance the diffusion and penetration of NPs within tissues, while conversely, lower temperatures may lead to aggregation and reduced bioavailability [141].

pH: The microenvironment surrounding NPs can exhibit significant variations in pH, influencing their surface charge and interactions with biological components [142]. In acidic environments, NPs may acquire a positive charge, promoting their interactions with negatively charged cell membranes and facilitating cellular uptake [143]. Conversely, in basic environments, NPs may exhibit neutral or negative charges, altering their interactions with cells and potentially hindering their therapeutic efficacy.

Oxygen Levels: Hypoxia, a state of limited oxygen availability, represents a significant challenge in diverse therapeutic applications, including tissue regeneration and cancer treatment [144]. Hypoxic conditions can impair cellular function and impede the effectiveness of conventional drug delivery systems (DDS) [136]. Moreover, DDS themselves can influence oxygen availability by altering the local microenvironment.

Biochemistry: The complex biochemical landscape surrounding NPs plays a crucial role in determining their biological interactions and fate. Biomolecules such as proteins, lipids, and nucleic acids can adsorb onto the surface of NPs, altering their physical and chemical properties and influencing their interactions with target cells or tissues [145,146]. Understanding the specific biochemical interactions occurring in the microenvironment is crucial for designing and optimizing NP-based therapies.

Pathogens and Toxins: The presence of pathogens or toxins can further complicate the biological journey of NPs [147]. Pathogens can compete with NPs for binding sites on cells, potentially hindering their therapeutic function [148]. Additionally, toxins released by pathogens can interact with NPs and alter their properties, potentially leading to unintended side effects.

Moisture: The absence of moisture can significantly impact the effectiveness of wound-healing therapies. Dryness can lead to cellular death chronic wound formation, and impede the epithelialization process. These factors limit the applications of conventional DDS for wound healing. Nanobiotechnology-based wound dressings offer a promising solution by providing sustained drug delivery and maintaining a moist environment at the wound site, promoting optimal healing conditions [149].

By understanding these complex interactions, researchers can design and develop next-generation NP-based therapies with tailored properties and controlled fates, paving the way for more effective and personalized treatment options in diverse biomedical applications.

## 12. Complications and Challenges

### 12.1. Cytotoxicity: Exploring Their Cytotoxic Effects on Non-Cancerous Cells

Nanoparticles (NPs), with their dimensions often less than 100 nanometers, have emerged as a revolutionary tool in medicine and various other fields. However, their extremely small size raises concerns regarding their potential to interact with and disrupt intracellular biochemical processes. This interaction can occur with various biological structures, including the cell wall, organelles, and even nucleic acids, potentially impacting the normal functioning of healthy cells [150,151].

- In vivo Concerns and the Uncertain Fate of NPs:

One of the most pressing concerns is the unknown fate of various nanobiotechnological products, particularly nanoparticles (NPs) of different sizes and materials. After entering the body, these NPs can interact with various biological components and distribute them to different organs. However, the precise pathways and long-term impact of this distribution remain largely unknown, creating uncertainty regarding their long-term effects on biological systems.

- Dose-Dependent Cytotoxicity: A Threat to Non-Cancerous Cells:

Several studies have demonstrated the dose-dependent cytotoxic effects of NPs on non-cancerous cells. For instance, AgNPs biosynthesized using Streptomyces sp. NH28 biomass exhibited a significant reduction in cell viability (82.9 ± 7.5%) in mammalian cells at concentrations as low as 25 µg/mL (IC50 64.5 µg/mL) [111,152]. Similarly, starch-stabilized AgNPs (20 nm) have been shown to decrease the viability of murine cells at concentrations of 10 µM [153].

These findings highlight the potential for unintended damage to healthy cells by NPs, even at relatively low concentrations. This raises concerns about the broader impact of such damage on organ function and overall health.

### 12.2. The Need for Comprehensive Research

To address these concerns and ensure the safe and responsible development of nanotechnology, comprehensive research is crucial. This research should focus on the following:

- Understanding the in vivo fate and distribution of NPs: This includes investigating their interactions with biological components, potential accumulation in organs, and long-term effects on health.
- Developing safe and effective NP designs: This includes optimizing the size, shape, and surface properties of NPs to minimize toxicity and maximize desired therapeutic effects.
- Evaluating the cytotoxicity and biocompatibility of NPs: This involves conducting rigorous studies to assess the potential harm of NPs on various cell types and tissues.
- Establishing regulatory frameworks: This includes developing guidelines and regulations for the development, testing, and use of nanomaterials to ensure safety and efficacy.

By addressing these concerns and conducting thorough research, we can unlock the vast potential of nanotechnology while minimizing potential risks and ensuring its responsible and beneficial development.

### 12.3. Impact of Functionalization and Material Composition

The biological actions and behavior of NPs are significantly influenced by various factors, including the following:

- Functionalization: Different functionalization approaches can alter the cytotoxicity and antibacterial properties of NPs. For example, SeNPs functionalized with poly-L-lysine (PLL) exhibited high levels of cytotoxicity and genotoxicity in TR146 (SCC), HaCaT, and Caco-2 cells, while PAA- and PVP-coated SeNPs showed no toxicity toward *E. coli*, *S. aureus*, or *S. cerevisiae* BY4741 [137].
- Materials used in fabrication: The specific material used to fabricate the NPs can also impact their cytotoxicity. For instance, commercially available AgNPs (15 and 100 nm) have been shown to induce significant toxicity in murine hepatocytes compared to NPs of manganese oxide, molybdenum, aluminum, iron oxide, or tungsten [154].

### 12.4. Functionalization: Tailoring the Nanoscale Armor

The remarkable potential of nanoparticles hinges heavily on their surface chemistry and the strategic placement of targeting ligands or antibodies [155,156]. These functionalities, often referred to as bioconjugates [157], determine the fate of the nanoparticles and their interactions with biological systems. Tailoring the surface properties of nanoparticles presents significant challenges, as the stability of the cargo, protection from enzymatic degradation, and evasion of the reticuloendothelial system (RES) must be meticulously considered [158].

Polymeric and organic nanomaterials offer remarkable advantages in this regard, readily accepting surface modifications with antibodies, peptides, or other biomolecules. This attribute enables them to efficiently encapsulate and deliver therapeutic agents or photosensitizers [159], paving the way for targeted therapies and diagnostics. Inorganic nanomaterials, on the other hand, pose greater challenges due to their inherent properties (e.g., fixed size, solid structure). While some can be surface-modified chemically or biologically, their limited cargo capacity restricts their functionality. Encapsulation with polymers, such as PEG or chitosan, offers a promising solution for inorganic nanoparticles, enabling them to carry biomolecules or drugs on their surface. However, the number and types of biomolecules that can be attached to the surface remain limited, governed by potential interactions and competition among them [160]. In contrast, liposomes, with their core–shell structure, offer the unique ability to simultaneously encapsulate a multitude of therapeutic or imaging agents, expanding the therapeutic potential of such systems [161].

### 12.5. Delivery and Targeting: Navigating the Biological Labyrinth

**Figure 7:**
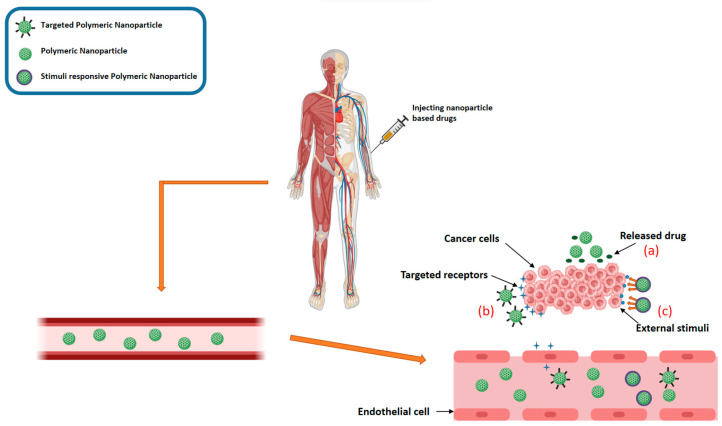
Representation showing various approaches to drug targeting: (a) Passive transport of nanocarriers through the permeable blood vessels of tumor tissue via extravasation; (b) Active targeted delivery to cancer cells; (c) Adaptive nanomedicines engineered to release anticancer agents in response to internal or external cues [168].

The intricate dance of delivery and targeting is influenced by a complex interplay of factors, including synthesis methods, functionalization strategies, local microenvironments, and administration routes [162]. Nanoparticles can reach their designated destinations through two main pathways: passive and active targeting (Figure 7) [163,164]. Passive targeting relies on the enhanced permeability and retention (EPR) effect, where nanoparticles exploit the leaky vasculature and impaired lymphatic drainage characteristic of tumor tissues. Conversely, active targeting leverages the power of specific ligands or antibodies that bind to unique receptors on target cells, enabling precise delivery and minimizing adverse effects on healthy tissues [165].

Despite the promising potential of active targeting, inherent challenges remain. Untargeted nanoparticles, lacking specific ligands or antibodies, are more susceptible to off-target diffusion, accumulating in healthy tissues and potentially inducing unwanted cytotoxicity [112,166]. Moreover, premature release of the therapeutic cargo or its degradation by the RES can significantly hinder the efficacy of nanoparticle-based therapies. Polymeric nanomaterials, liposomes, and micelles, with their well-defined structures and encapsulation capabilities, offer an advantage in this regard, providing greater protection and controlled release of therapeutic agents [167].

Unfortunately, the intricate interplay between the tumor microenvironment (TME) and intracellular biochemistry adds another layer of complexity to the delivery process. Factors such as cell type, disease state, and intercellular interactions can significantly influence the behavior of nanoparticles, making it challenging to predict their precise delivery and therapeutic outcomes. Therefore, meticulous investigation and thorough characterization are essential to fully understand the delivery capacity and biochemical interactions of drug-loaded nanoparticles within diverse biological systems.

### 12.6. The Influence of Size and Physical Parameters on Biological Fate

The intricate relationship between the physical characteristics of nanoparticles (NPs) and their biological impact is a complex dance with far-reaching consequences. Size, in particular, plays a pivotal role in shaping the fate of NPs within mammalian cells.

#### 12.6.1. Size-Dependent Plasmon Absorption and Biological Interactions

The size of NPs significantly influences their biological interactions with cellular structures. This phenomenon is attributed to the size-dependent dielectric function of NPs, which governs their plasmonic resonance (SPR) behavior [169]. SPR is the collective oscillation of electrons in the NP’s conduction band, and it is highly sensitive to particle size and shape. As a result, any shift or fluctuation in the SPR band can significantly impact the absorption bandwidth of light, ultimately influencing how NPs interact with biological structures [170].

#### 12.6.2. Beyond Size: The Symphony of Geometry, Topography, and Zeta Potential

While size plays a crucial role, other physical parameters like geometry, topography, and zeta potential (ZP) also contribute to the intricate biological interactions of NPs. The geometry of NPs, encompassing their shape and surface features, can influence factors like cellular uptake, intracellular trafficking, and overall biological response [171]. Additionally, the topographical features of NPs, such as surface roughness and porosity, can further influence their interactions with biological structures [172].

### 12.7. The Complex Orchestration of Biological Fate

Their physical characteristics do not solely determine the biological fate of NPs. The local environment, the presence of targeting ligands, and the type of nanomaterial all play crucial roles in dictating their interactions with biological systems. However, despite these confounding factors, the physical parameters and dimensions of NPs serve as a fundamental layer that significantly influences their interactions and ultimately shapes their biological fate.

### 12.8. Beyond SPR: Modulating Biological Fate through Physical Features

The influence of physical parameters on biological fate extends beyond SPR. For instance, size can impact the ability of NPs to cross biological barriers and penetrate specific tissues [173]. Similarly, the geometry and surface properties of NPs can influence their interactions with specific cellular receptors, leading to targeted delivery or enhanced cellular uptake [174,175].

By understanding the intricate interplay between the physical characteristics of NPs and their biological impact, researchers can design and develop next-generation nanomaterials with tailored properties and functionalities. This knowledge holds immense promise for revolutionizing diverse fields, including drug delivery, regenerative medicine, and cancer therapy.

## 13. Future Perspective: Unveiling the Untapped Potential of Nanobiotechnology

Despite facing challenges and limitations, nanobiotechnology holds immense promise for delivering high-quality therapeutics with unparalleled precision and predictability. By delving deeper into its diverse branches, like immunotherapy and gene therapy, researchers can address existing bottlenecks and unlock new avenues for healthcare advancements. The rapid pace of progress in nanobiotechnology, fueled by cutting-edge research across academia and industry, signifies its potential to revolutionize healthcare through the development of novel methods, techniques, and materials for theranostics [176,177].

While the applications of nanomaterials and other nanobiotechnological products are vast, it is crucial to prioritize those that offer the most significant benefits. This requires focusing on developments that enhance efficiency, improve scientific understanding, and ultimately contribute to an overall improved quality of life. A deeper understanding of the interactions between nanomaterials and biological systems, including organelles, ecological environments, and animal models, is vital for the responsible and ethical development of this transformative technology.

Here are some key areas where nanobiotechnology is expected to make significant contributions in the future:

- Precision Medicine: Nanobiosensors and nanocarriers will enable the development of personalized therapies tailored to individual patients’ genetic profiles and disease states. This personalized approach holds the promise of improving treatment efficacy and reducing side effects [178].
- Regenerative Medicine: Nanobiotechnology offers exciting opportunities for tissue regeneration and repair. Nanoparticles and scaffolds can be used to deliver stem cells and other therapeutic agents to damaged tissues, promoting healing and restoring function [179].
- Drug Delivery: Nanocarriers can be designed to deliver drugs specifically to diseased tissues, minimizing off-target effects and improving therapeutic efficacy. Additionally, stimuli-responsive nanocarriers can release their cargo in response to specific triggers, further enhancing targeted delivery [180].
- Diagnostics: Nanobiosensors offer high sensitivity and specificity for disease detection, allowing for early diagnosis and intervention. This can lead to improved patient outcomes and reduced healthcare costs [181].
- Environmental Remediation: Nanomaterials can be used to remove pollutants from water and soil, contributing to a cleaner and healthier environment. Additionally, nano biosensors can detect environmental contaminants at very low levels, enabling early intervention and remediation [182].
- Food and Agriculture: Nanobiotechnology can be used to improve food production and safety. Nanoparticles can be used to deliver nutrients to plants and protect them from pests and diseases. Additionally, nano biosensors can detect foodborne pathogens and contaminants, ensuring the safety of the food supply [183].

Nanobiotechnology is poised to revolutionize various fields, from healthcare and medicine to environmental remediation and agriculture. By addressing existing challenges and pursuing responsible development, this transformative technology holds the potential to improve the quality of life and create a healthier planet for future generations.

## 14. Conclusions

In conclusion, the transformative impact of nanobiotechnology on healthcare is undeniable, offering unprecedented opportunities for precise and effective therapeutic delivery. The ability to tailor nanocarriers at the molecular level through nanoscale manipulation empowers researchers to optimize size, shape, and surface properties, enhancing therapeutic outcomes. Polymer nanocarriers, with their structural versatility and unique properties, stand out as compelling options, allowing for efficient and versatile delivery systems. Targeted and triggered release strategies further enhance precision, ensuring therapeutic agents reach specific tissues while minimizing side effects. Nanobiotechnology also unlocks the therapeutic potential of phytochemicals, addressing issues of poor solubility through controlled delivery. As we look to the future, the horizon of possibilities in nanobiotechnology is vast, with ongoing research poised to revolutionize healthcare through the development of more efficient, targeted, and personalized therapeutic approaches. Continued exploration of novel methods, nano-biomaterials, and devices will undoubtedly propel nanobiotechnology into new frontiers, addressing challenges and realizing its full potential in shaping the future of healthcare.

## Figures and Tables

**Table 1:** Nanostructure types and their biomedical applications [15,16,18].

Type of Nanostructure	Material Composition	Biomedical Application	Advantages	Challenges
Liposomes	Lipid bilayers	Drug delivery	Biocompatible drug encapsulation	Limited stability
Polymeric Nanoparticles	Synthetic polymers	Targeted drug delivery	Controlled release, versatility	Potential toxicity
Micelles	Amphiphilic molecules	Drug delivery, imaging	Enhanced solubility, stability	Limited cargo capacity
Dendrimers	Branched macromolecules	Drug delivery, diagnostics	Precise structure, multivalency	Complex synthesis, potential toxicity
Quantum Dots	Semiconductor nanocrystals	Imaging, diagnostics	Bright fluorescence, tunable size	Heavy metal toxicity
Gold Nanoparticles	Metallic gold	Imaging, therapy	Surface plasmon resonance, biocompatibility	Clearance concerns
Iron Oxide Nanoparticles	Magnetic iron oxide	Imaging, hyperthermia	MRI contrast, magnetic targeting	Biodegradability
Carbon Nanotubes	Carbon allotropes	Drug delivery, imaging	High aspect ratio, electrical conductivity	Biocompatibility concerns
Silica Nanoparticles	Silicon dioxide	Imaging, drug delivery	Biocompatible, versatile surface modification	Degradability concerns

**Table 2:** Biocompatible and Biodegradable Nanoparticles for Drug Delivery: A Comparison of Polymeric Nanoparticles (ChNPs, PEG, PLA, PCL, PAA) and Lipid-based Nanoparticles (Liposomes, Nanoliposomes) [55,56,57].

Feature	Polymeric NPs (ChNPs, PEG, PLA, PCL, PAA)	Lipid-Based NPs (Liposomes, Nanoliposomes)
Synthesis complexity	Often requires multiple chemicals/solvents, potentially raising environmental concerns.	Easier formulation; thin-film dispersion technique commonly used.
Biocompatibility	It may need capping with polymers/peptides (PEGylation) for improved biocompatibility.	Inherently biocompatible
Drug compatibility	Suitable for both hydrophobic and hydrophilic drugs.	Ideal for both hydrophobic and hydrophilic drugs.
Targeting potential	Limited; recent advancements in nanoliposomes with mannose–cholesterol conjugation show promise.	High surface modifications allow for the incorporation of target ligands.
Versatility	It can be tailored in size and composition but has limited capacity for multidrug encapsulation.	Highly versatile; can be tailored in size, composition, and lipid molecules; can encapsulate multiple drugs, contrast agents, and photosensitizers.
Environmental impact	It can involve harsh chemicals and solvents.	Potentially more environmentally friendly
Examples of successful applications	ChNPs: Gene delivery, cancer therapy	Liposomes: Doxorubicin delivery for cancer, brinzolamide delivery for glaucoma.
Future potential	Exploring more eco-friendly synthesis methods; the need for improved targeting strategies.	Optimizing targeted delivery, developing multi-functional liposomes for sequential release.

**Table 6:** Nanoparticles in Cancer Therapy [111,112,113,114].

Nanoparticle Type	Targeted Cancers	Mechanism of Action	Advantages	Disadvantages	Examples in Clinical Use
Liposomes	Breast, lung, ovarian, leukemia	Drug delivery, sustained release, improved biocompatibility	Enhanced drug targeting, reduced side effects	Potential leakage of the drug, difficulty in scale-up production	Doxil^®^ (doxorubicin), Abraxane^®^ (paclitaxel)
Polymeric Nanoparticles	Brain, pancreatic, colorectal, melanoma	Drug delivery, gene therapy, imaging	High drug loading capacity, versatile surface modification	Potential toxicity of some polymers, degradation concerns	Oncaspar^®^ (L-asparaginase), Campath^®^ (alemtuzumab)
Gold Nanoparticles	Breast, prostate, ovarian, head, and neck	Photothermal therapy (PTT), photodynamic therapy (PDT), imaging	High heat conversion efficiency, targeted delivery	Potential cytotoxicity, long-term safety concerns	AuroShell^®^ (breast cancer), Aurinocle^®^ (melanoma)
Iron Oxide Nanoparticles	Liver, brain, lymph nodes	Magnetic resonance imaging (MRI), hyperthermia therapy	High specificity, low toxicity	Potential aggregation, limited drug loading capacity	Feridex^®^ (liver imaging), NanoTherm^®^ (tumor ablation)
Carbon Nanotubes (CNTs)	Lung, brain, ovarian, leukemia	Drug delivery, gene therapy, photothermal therapy	High surface area, tunable properties	Potential off-target effects, safety concerns	BIND Therapeutics (lung cancer), CTI BioPharma (liver cancer)
Mesoporous Silica Nanoparticles (MSNs)	Lung, breast, colon, melanoma	Drug delivery, controlled release, imaging	High drug loading capacity, biocompatible	Potential pore blockage, difficulty in surface modification	STARpharma (breast cancer), OncoSil (prostate cancer)
Quantum Dots (QDs)	Brain, lymph nodes, tumors	Imaging, biosensing	High brightness, multiplexing capability	Potential toxicity of heavy metals, biodegradation concerns	Qdot^®^ (tumor targeting), Icosagen (lung cancer)

**Table 7:** Comprehensive overview of the various aspects of cellular disruption and therapeutic potential associated with nanoparticles.

Nanoparticle Types	Description
Polysaccharides for Dermatological and Photodynamic Therapy	Polysaccharides are biocompatible and biodegradable natural polymers with applications in dermatology and photodynamic therapy (PDT). In dermatology, they are used as hydrogels for drug delivery, wound healing, and cosmetics. In PDT, they serve as carriers for photosensitizers that generate reactive oxygen species (ROS) to target cancer cells or bacteria upon light activation. Their functional groups enable drug conjugation, targeting, and imaging.
Glycopolymer-Functionalized Nanoparticles for Targeted Delivery	Glycopolymer-functionalized nanoparticles are nanoparticles modified with glycopolymers, which have sugar molecules that can target specific cell receptors. This makes them ideal for targeted drug delivery, especially for targeting cancer or diseased cells. Once they bind to the target cells, they release the drug inside, where it can take effect.
Doxil and Caelyx	Doxil and Caelyx are liposomal formulations of the chemotherapy drug doxorubicin, designed to reduce side effects like cardiotoxicity and hair loss by targeting cancer cells more precisely. They are used to treat cancers such as ovarian cancer, breast cancer, and Kaposi’s sarcoma.
Liposomes	Liposomes are artificial vesicles composed of a phospholipid bilayer, similar to a cell membrane. This structure enables them to fuse with cells and deliver drugs, including anticancer agents, antibiotics, and gene therapy vectors. They are also being explored for imaging applications like MRI and ultrasound.
Expanding the Repertoire of Nano-Drugs	Nanomedicine is rapidly advancing, with many nano-drugs in development. These drugs, including antibody-drug conjugates, siRNA nanoparticles, and polymeric micelles, target diseases at the molecular level, offering more effective and less toxic treatments.
MNPs: Cancer Therapy	Magnetic nanoparticles (MNPs) are tiny particles with a magnetic core and a non-magnetic coating. The magnetic core enables them to be controlled by an external magnetic field. This feature is particularly useful in cancer therapy, where MNPs can be directed to tumors and heated to destroy cancer cells. They are also being explored for drug delivery and imaging applications.
